# MRI safety in patients with abandoned temporary epicardial pacing wires: an eight-year retrospective study: Original Research Article

**DOI:** 10.1007/s10554-025-03482-y

**Published:** 2025-08-07

**Authors:** Jordan H. Chamberlin, Eleanor Glenn, Fatemeh S. Hamishegi, Jeremy R. Burt, Michael Antonucci, Dhiraj Baruah, Ismail Mikdat Kabakus

**Affiliations:** 1 Division of Cardiothoracic Imaging, Department of Radiology, Medical University of South Carolina, Charleston, SC USA; 2 Faculty of Medicine, Guilan University of Medical Sciences, Rasht, Iran; 3 Division of Cardiothoracic Imaging, Department of Radiology, University of Utah, Salt Lake City, UT USA; 4 Division of Neuroradiology, Department of Radiology, Medical University of South Carolina, Charleston, SC USA

## Abstract

To evaluate the prevalence and types of adverse events associated with magnetic resonance imaging (MRI) in patients with abandoned temporary epicardial pacing wires (TEPW) over an eight-year period. A total of 54 patients and 104 MRI examinations with abandoned TEPW (9 cardiac, 6 non-cardiac thoracic, 89 other) were included in the final analysis. Imaging was reviewed to confirm wire presence and billing information was queried to identify hardware manufacturer. The prevalence of major and minor adverse events as clinically documented or reported by the MRI safety team was recorded. Results were contextualized based on the duration that wires were in situ, when this information was available. No major adverse events were recorded. One minor sensory event occurred during a 1.5T cervical spine MRI in a patient with wires retained for 215 days. No acute abnormalities or wire positioning changes were noted. In situ duration ranged from 3 to 8684 days, with 4.8% of patients having wires for less than 1 month. Brain (36%) and abdominal (28.2%) MRIs were most frequent. No adverse events were reported in cardiac or thoracic MRIs. Most leads, including in the minor event, were Medtronic Bipolar Coaxial 6495. MRI in patients with abandoned TEPW has a very low rate of minor adverse events. No major events occurred over 8 years. MRI should not be withheld from patients with abandoned TEPW. Routine discussion of risks and benefits should continue with recognition that alternative tests may obviate the need for MRI. The low prevalence of minor adverse events is reassuring, and risks can be mitigated by ensuring compliance with manufacturer guidelines and minimizing energy deposition during imaging.

## Introduction

Temporary epicardial pacing wires (TEPW) are utilized after cardiac surgeries to manage arrhythmias and facilitate the transition from cardiopulmonary bypass pump to normal cardiac activity and are placed on the ventricles, atria, or both [1, 2]. Typical manufacturer guidelines state leads should be fully removed within seven days post-operation by gentle traction [3, 4]. However, TEPW or their fragments may be retained in the patient’s body due to unsuccessful extraction or clipping at the subcutaneous tissues. Uncommon but serious complications (i.e. cardiac tamponade, ventricular arrhythmia) exist with removal of the pacing wires and as a result, clipped and abandoned leads are becoming more common [1, 4]. Clipped abandoned fragments are unlikely [5] to result in serious clinical complications [3, 6], but may pose a potential magnetic resonance imaging (MRI) safety risk due to the conductive nature of the leads.

Epicardial pacing wires are used to deliver electrical impulses to the heart to manage arrhythmias or support cardiac function, typically post-cardiac surgery [4, 7]. They are classified into temporary and permanent types, each with distinct purposes, designs, and MRI safety considerations [8]. Based on theoretical considerations, abandoned permanent or temporary abandoned pacing wires/wire can be safety risk for MRI. MRI may cause electromagnetic interference in the permanent or temporary pacing wires, potentially leading to arrhythmia or thermogenic damage due to the heat discharged from the affected wires [4, 9–13]. Despite long-standing safety concerns, studies have demonstrated no major complications in patients with abandoned permanent or temporary pacing wires [14–18]. Hartnell et al. reported no major adverse events in 51 patients with abandoned TEPW who underwent MRI at 1–1.5T [15]. In a study by Pfeil et al. using an ex vivo pig heart model, no histopathological signs of thermal damage were observed around the tips of the temporary epicardial pacing leads in any of the hearts [18]. Ma et al. analyzed a total of 29 patients with permanent epicardial leads, comprising both active (55%) and retained (45%) leads, to assess major and non-major complications after undergoing MRI. Zero major adverse events, lead dysfunction, or heating damage were reported during the follow-up [19].

American College of Radiology Manual on MR Safety 2024 Update specifically notes that no adverse outcomes have been reported to date in association with retained temporary epicardial wire fragments. It also clarifies that postsurgical temporary epicardial wires that have been partially removed are not classified as abandoned pacing leads [8]. Despite the growing body of promising data supporting MRI safety in patients with abandoned TEPW, their safety remains a subject of ongoing debate. Some clinicians, in the absence of evidence, may overestimate the potential for adverse events, leading to unnecessary avoidance of critical imaging. As a result, patients with TEPW may be excluded from MRI examinations, potentially delaying essential diagnostic evaluations.

While data has been promising, the available information is only in small sample sizes and often only amongst a narrow range of MRI examinations. Therefore, the purpose of this study is to present a longitudinal evaluation of MRI safety in patients with specifically temporary epicardial pacing wires across a broad selection of pertinent examinations. The authors hypothesize that the rate of adverse events related TEPW will be low and that the results will support the safe use of MRI in patients with TEPW.

## Materials and methods

### Subjects and ethics statement

This single institution, retrospectively performed, observational cohort study was approved by the Institutional Review Board at the performing institution (Pro00135217), and the need for written informed consent was waived. Patient data, including identifiers, were recorded and stored in compliance with the Health Insurance Portability and Accountability Act in an encrypted web database in institutional storage. Patient data was de-identified prior to statistical analysis, and no individual patient information was shared with third party entities.

**Fig. 1:**
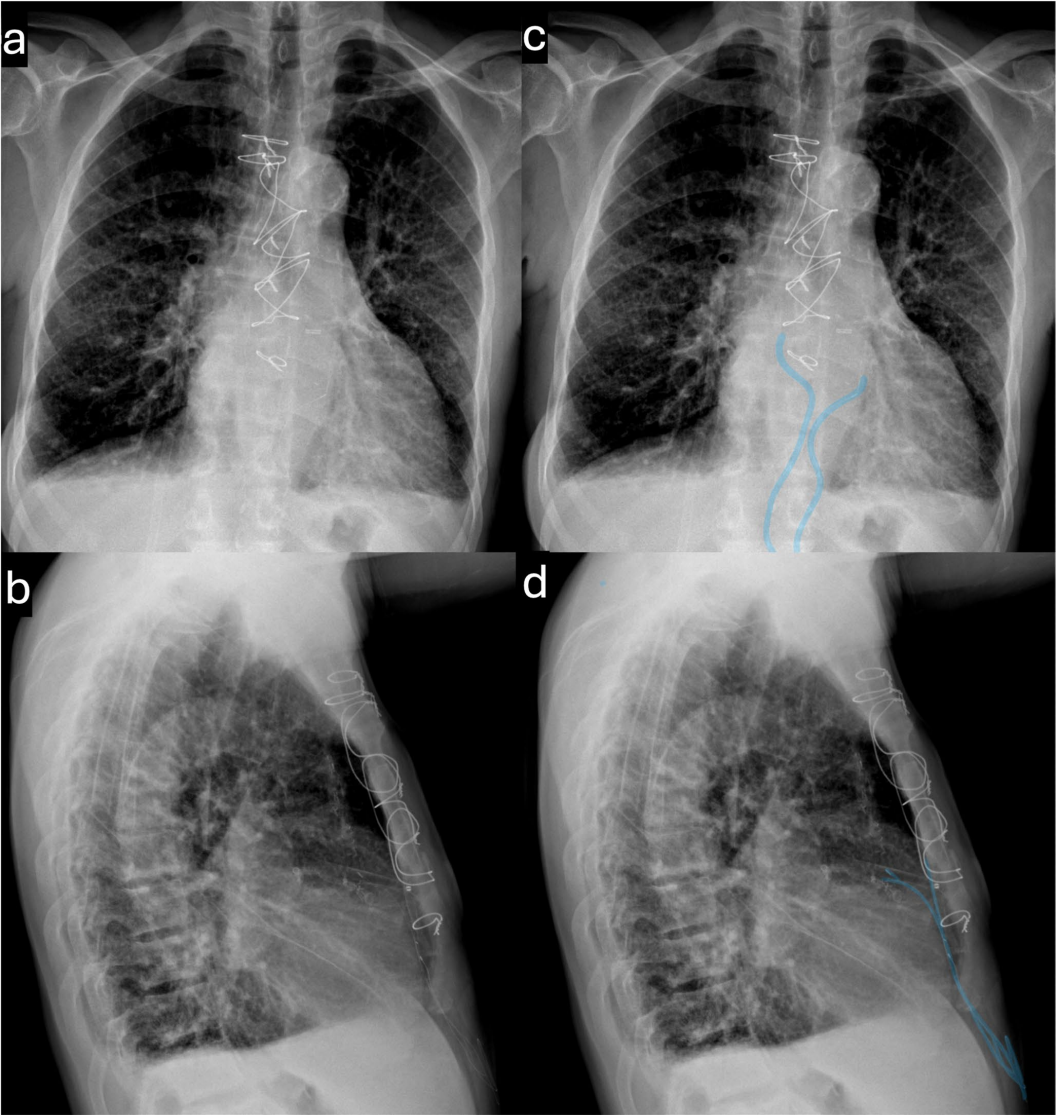
Chest X-rays displaying abandoned epicardial pacing wires. Panels a and b show the frontal and lateral views, respectively, without annotations. Panels c and d highlight the epicardial pacing wires, marked with blue lines, on the corresponding frontal (c) and lateral (d) chest X-rays

**Fig. 2:**
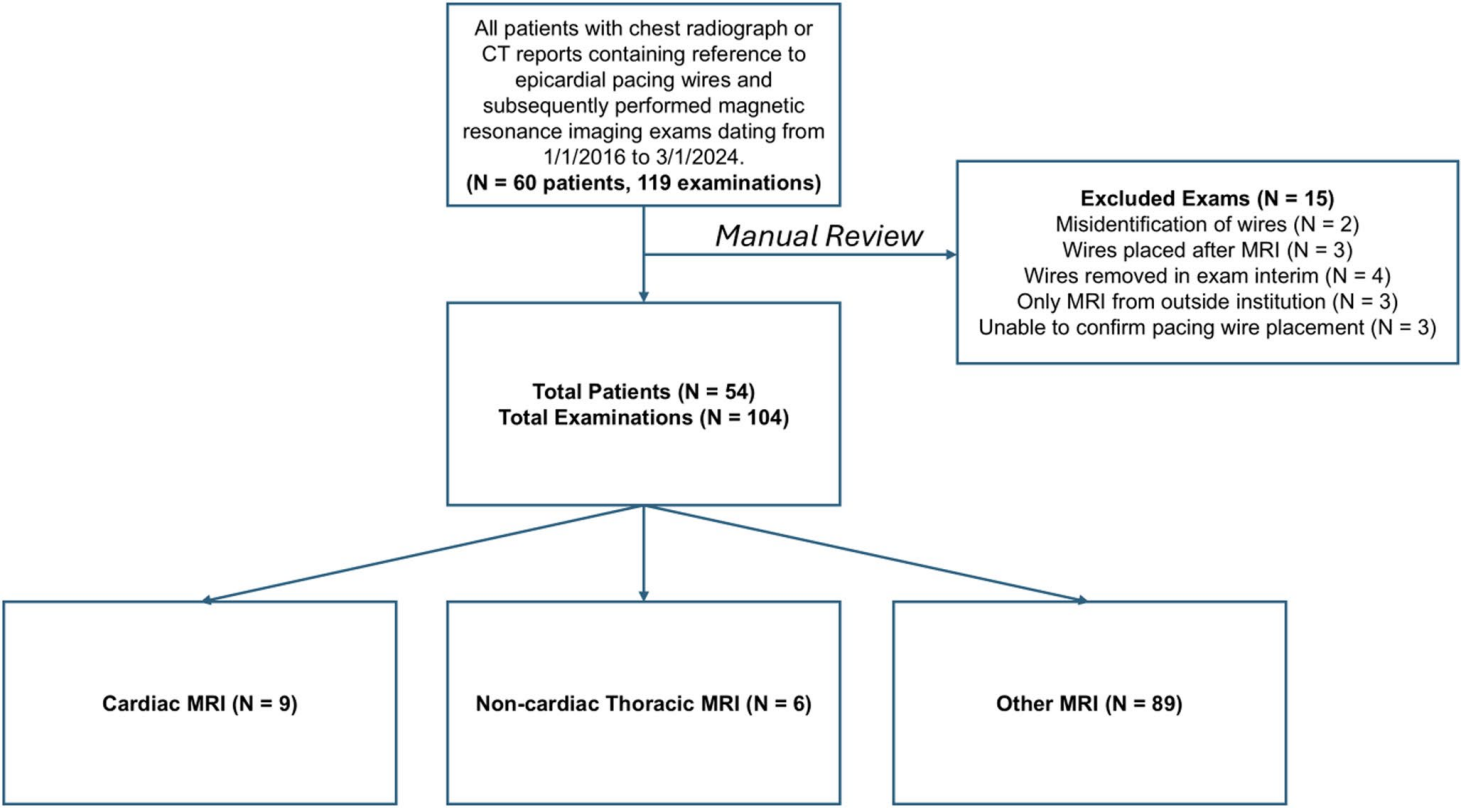
Inclusion flowchart

Inclusion criteria included patients > 18 years of age with temporary epicardial pacing wires present at the time of magnetic resonance examination between 1/1/2016 and 3/1/2024. Exclusion criteria included misidentification/labeling of TEPW, TEPW placed after the MRI or removed in the interim, examinations from outside institutions without accompanying clinical details, and inability to confirm epicardial wire placement. Patients were identified through a comprehensive search of patient charts and the radiology report database using the keyword “epicardial pacing wires.” Confirmation of the presence of TEPW on imaging was performed by a cardiothoracic radiologist with more than 10 years of experience (Fig. 1**).** Final inclusion criteria yielded 54 patients with abandoned epicardial pacing wires who underwent a total of 104 MRI examinations. Nine cardiac MRIs, 6 non-cardiac thoracic MRIs, and 89 other MRIs were included. A study flowchart is given in Fig. 2.

### Data collection

Basic patient demographics and diagnoses were documented including age and sex. Race/ethnicity data was deemed not to be pertinent and therefore not recorded. The manufacturer make and model of the TEPW were recorded when this information was available in the patient charts, particularly for pacing wires placed at our institute or when outside records were available. During each MRI encounter, any adverse events were recorded as part of routine documentation attached to the examination order. Life-threatening events, such as cardiac arrhythmia, cardiac arrest, and major burns causing irreversible damage, were classified as major adverse effects. Minor events were defined as non-life-threatening adverse events including but not limited to mild thermal heating, unusual sensations, or pain. When knowledge of the epicardial pacing wires was available, standard MRI safety procedures and patient informed consent were obtained in accordance with institutional policy. In some urgent cases, the presence of epicardial pacing wires was not checked prior to the MRI, resulting in some scans being performed on a 3 T MRI scanner.

### Examination grouping and acquisition protocols

104 MRI examinations were categorized into three groups based on the region of interest:

Cardiac magnetic resonance (CMR), thoracic MRI and other (all remaining studies). Details such as examination date and magnet field strength were recorded. No information regarding contrast administration or specific sequences was routinely recorded as examinations were extremely heterogenous. All examinations were performed as routine protocol dictated by institution policy, plus or minus additional sequences based on provider or radiologist preference.

At our institution, when scanning patients with retained epicardial pacing wires, we adhere to the same safety precautions used for patients with MRI-conditional devices. A dedicated MRI physicist and a nurse are always present during the scan, continuously monitoring the patient throughout the procedure. This includes real-time assessment of vital signs and communication with the patient to detect any discomfort or abnormal sensations. Additionally, lower-strength MRI, such as the 1.5 Tesla, is used, with scanning protocol and parameters adjusted when necessary to minimize potential risks. This includes reducing the specific absorption rate or modifying sequence protocols to further enhance patient safety. These measures ensure that all appropriate safety standards are met during the MRI examination.

### Cardiac devices

Three different TEPW models were positively identified. Medtronic (Minneapolis, MN) Bipolar Coaxial model 6495 were the most frequently identified. Per manufacturer specifications the device “is intended for temporary postsurgical atrial and ventricular pacing and sensing for a contemplated implant duration of 7 days or less. The device is supplied sterile and is intended for single use only (https://www.medtronic.com/us-en/healthcareprofessionals/products/cardiovascular/heart-valves-surgical/temporary-myocardial-pacing-leads/indications-safety-warnings.html). No specific MRI recommendations are given by the manufacturer. One patient with an unspecified Abbott Cardiovascular (Plymouth MN) retained TEPW was included. Per manufacturer information, specific Abbott temporary pacing wires may be MRI conditional, but definitive identification was not possible (https://manuals.eifu.abbott/en/hcp/home.html). A single St. Jude Medical Myodex 1084 T TEPW was identified. (Little Canada, MN; now Abbott Cardiovascular; https://www.cardion.cz/file/1025/myodex-specsheet.pdf). No specific MRI compatible recommendations are available for this device.

### Statistical analysis

The primary outcome of this study was defined to be the prevalence of adverse events. Therefore, no statistical tests were performed, and a power analysis was therefore omitted. Data was reported with medians and interquartile ranges for continuous variables and count plus frequency for categorical variables. Time interval was defined as the duration between TEPW placement and examination date. In the event that the precise date of the placement was unclear or incomplete (e.g., records indicating cardiothoracic surgery “May 2024”), the first of the month or year was recorded (e.g., 1/1/2024 in the event of year only or 5/1/2024). This approach was chosen as there would be less bias regarding early TEPW placement and possible adverse events in the early post-operative period.

## Results

Between 1/1/2016 and 3/1/2024, 60 patients and 119 MRI examinations were identified using clinical database search queries for “epicardial pacing wires”. A total of 54 patients and 104 MRI with TEPW (9 cardiac MRI, 6 non-cardiac thoracic MRI, 89 other) examinations met the final inclusion criteria **(**Fig. 2**).** The median patient age was 47 ± 14 years for cardiac MRI, 70 ± 16 years for non-cardiac thoracic MRI, and 64 ± 26 years for all other MRI examinations. There was a male predominance for cardiac MRI (*N* = 6, 66.7%) and all other MRI (*N* = 61, 68.5%). Three manufacturers were identified: Abbott Cardiovascular - Plymouth MN, Medtronic– Minneapolis MN, and St. Jude Medical (prior to acquisition by Abbott). By far the most frequent TEPW was the Medtronic Bipolar Coaxial 6495 which was identified 55 times (61.8%). 32 (36%) patients did not have brand identification. The most used MRI field strength was 1.5 Tesla (*N* = 94, 90.3%).

**Fig. 3:**
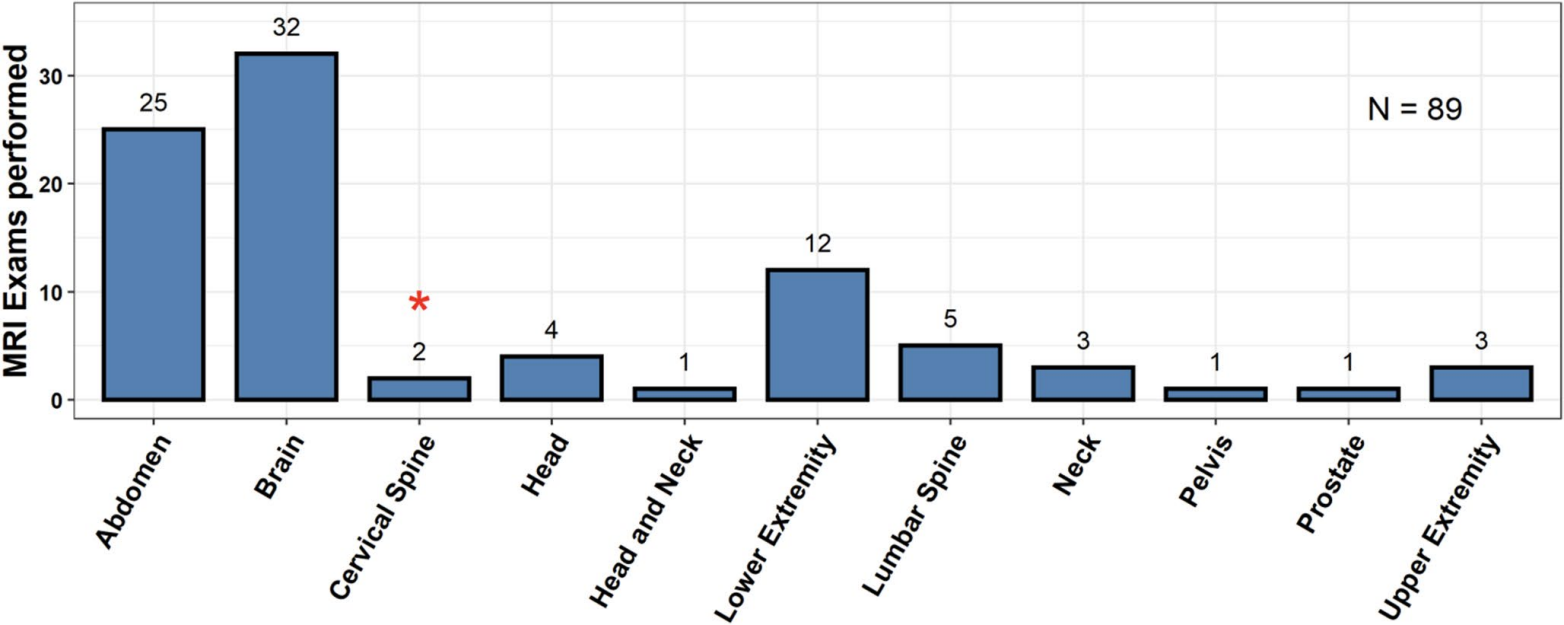
Non-cardiac or thoracic MRI examinations included in the final analysis. Abdominal and brain MRIs were the most frequently performed examinations followed distantly by lower extremity MRIs. The minor event was recorded during an examination of the cervical spine as demarcated by the red asterisk

**Fig. 4:**
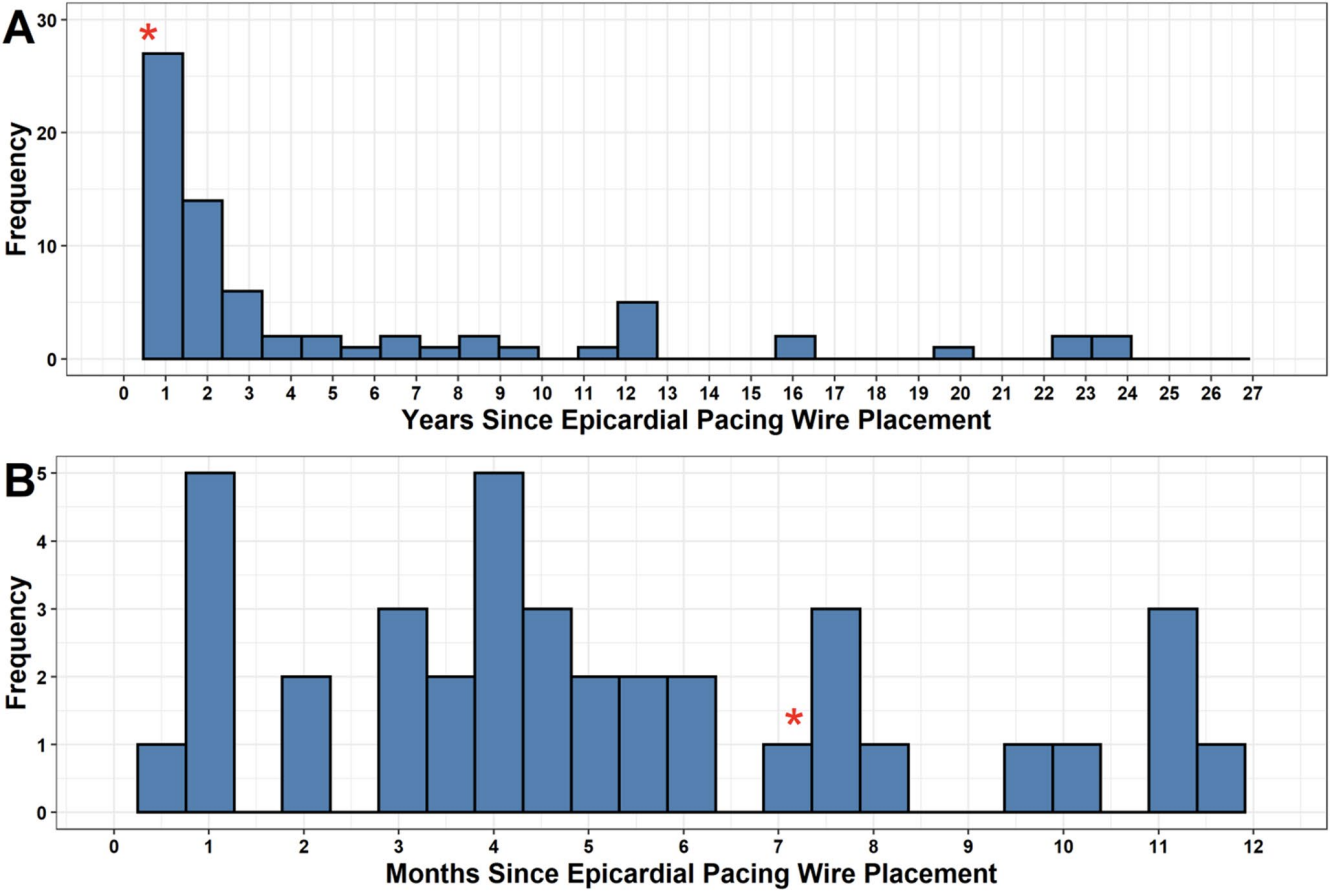
Distribution of the time in situ for TEPW. **(A)** The majority of examinations were performed in the first two years trailing off through the first decade. Scattered examinations were performed up to nearly 24 years post-implantation. **(B)** Examinations during the first year mostly occurred for 3–6 months post-placement. There was a minority of examinations performed in the first month (*N* = 5, 4.8%). The examination associated with a minor adverse event occurred 215 days post implantation (~ 7 months) as denoted by the red asterisk

The median time in-situ was longest for patients undergoing cardiac MRIs (2302 ± 4322 days). Non-cardiac thoracic MRI time in situ was shorter with a median of 469 ± 397 days. All other MRIs were similar, with a median time in situ of 444 ± 970 days. The range of days was 3–8684 days (23.4 years) **(**Table 1**).** The composition of other MRIs was diverse. Abdominal (*N* = 25, 28.1%) and Brain MRIs (*N* = 32, 36.0%) were the most frequently performed examinations. **(**Fig. 3**).** TEPW had a diverse time in situ. The majority of TEPW had been in place between 1 and 2 years, but a significant proportion were in place for up to 10 years with a few patients having TEPW in situ for over 15 years. Few examinations were performed in the immediate post-operative period, with only 5 (4.8%) occurring at or before one month **(**Fig. 4**).**

**Table 1:** Summary data

*N* = 54 Patients, 104 Exams	Cardiac MRI (*N* = 9)	Non-Cardiac Thoracic MRI (*N* = 6)	Other MRI (*N* = 89)
	Median (IQR)| N (%)		
Age at MRI (years)	47 (14)	70 (16)	64 (26)
Sex Female Male	3 (33.3) 6 (66.7)	3 (50) 3 (50)	28 (31.5) 61 (68.5)
TEPW Manufacturer Abbott Medtronic St Jude Unknown	0 (0) 3 (33.3) 0 (0) 6 (66.7)	0 4(66.7) 0 2(33.3)	1 (1.1) 55 (61.8) 1 (1.1) 32 (36.0)
TEPW Model Unspecified Abbott Medtronic Bipolar Coaxial 6495 St Jude Myodex Bipolar IS	0 3(33.3) 0	0 4(66.7) 0	1 (1.1) 55 (61.8) 1 (1.1)
TEPW Placement– MRI interval (days)	2302 (4322)	469 (397)	444 (970)
MRI Field Strength (T) 1.5 3.0	8 (88.9) 1 (11.1)	5 (83.3) 1 (16.7)	81 (91.0) 8 (9.0)
Minor Complications	0	0	1
Major Complications	0	0	0

**Fig. 5:**
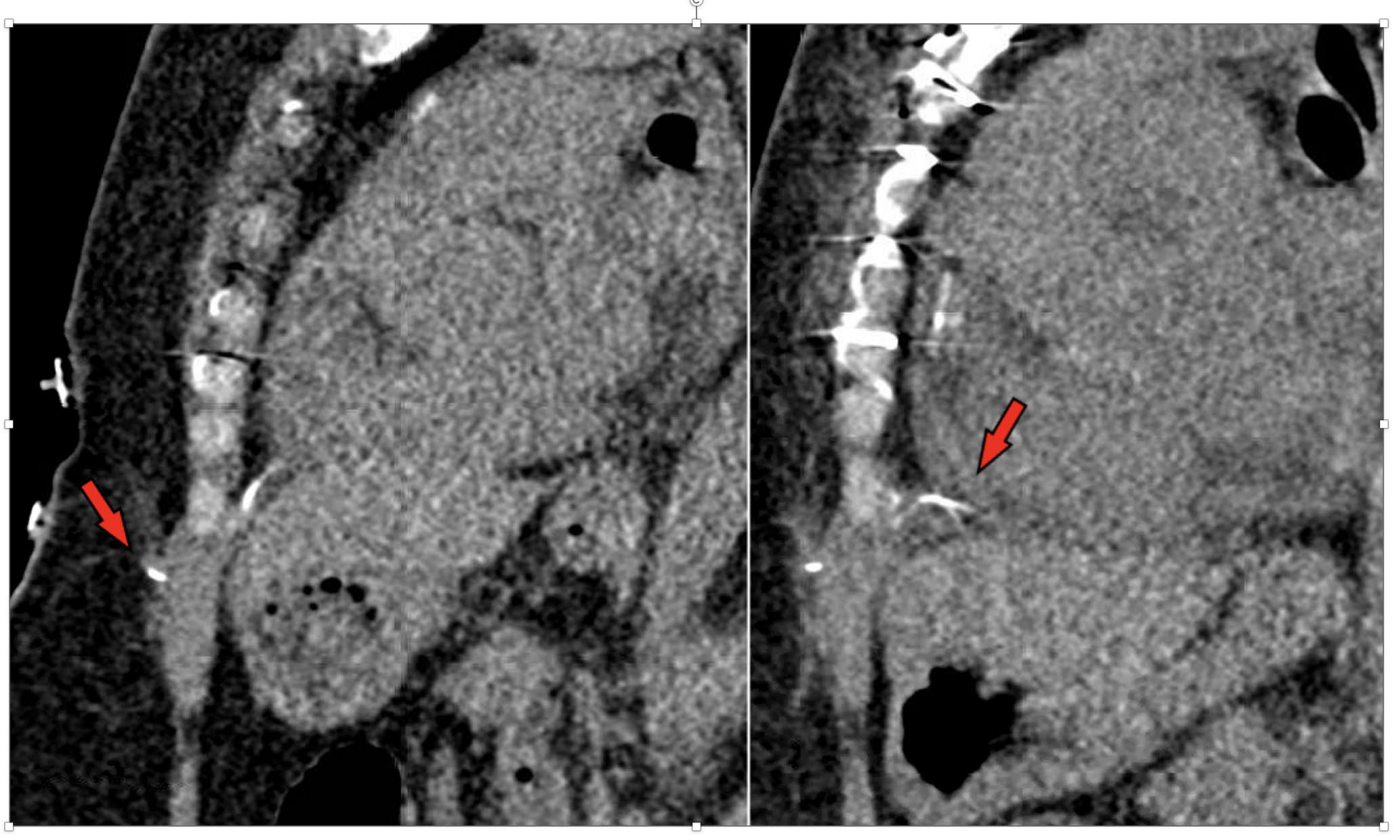
Non-contrast chest CT sagittal reconstructions for the patient case with minor skin sensation reaction during cervical spine MRI. Arrows demarcate the truncated epicardial pacer lead in the subxiphoid soft tissues and terminal abutting the anterior heart (red arrows). Partially visualized additional epicardial pacing wires over the right atrium. There are expected post-surgical changes about the anterior body wall. No fluid collection or reactive changes in the soft tissue are noted. There was no pericardial effusion. The leads were noted to be in an unchanged position in comparison with a prior chest CT

There was a single identified minor adverse event (prevalence of 0.9%) and zero major adverse events. The event occurred during a 1.5T cervical spine MRI in an inpatient with abandoned Medtronic Bipolar Coaxial 6495 TEPW in situ for 215 days. The minor adverse event was characterized as a subjective burning sensation over the chest immediately after the localizer sequence, during the acquisition of sagittal T2-weighted turbo spin echo sequence. The examination was immediately ended, and cardiology team was consulted. Physical examination and electrocardiogram findings were unchanged from baseline. A transthoracic echocardiogram was unremarkable for acute findings or pericardial effusion. A CT chest was performed, which showed expected post-operative changes in the subxiphoid soft tissues without acute intrathoracic etiology or fluid collection. The CT chest confirmed placement of the TEPW perpendicularly oriented to the chest wall with terminal ends adjacent to the anterolateral left ventricle and right atrium. There was no looping or adjacent metallic material other than median sternotomy wires, which were intact and collinear. The patient had two abandoned epicardial pacing wires, each approximately 15 cm in length. The patient did not have permanent pacemaker leads. **(**Fig. 5**).**

## Discussion

The purpose of this study was to evaluate the prevalence and characteristics of adverse events associated with abandoned TEPW at a tertiary center over an eight-year period of time. The results demonstrate a low rate of adverse events associated with abandoned TEPW. Only one minor sensation event was recorded in the 8 years where detailed electronic medical record information was available, adding weight to the prior literature [8, 20, 21]. The patients underwent a variety of magnetic resonance examinations, including dedicated cardiovascular MRI. Interestingly, there was no correlation between the dedicated cardiac or thoracic examinations and adverse events as initially hypothesized, as the sole minor event occurred during a cervical spine MRI. Furthermore, neither manufacturer, time in situ, nor magnetic field strength was obviously correlated. Most importantly, no major events were recorded over nearly a decade, suggesting that major events are a very rare phenomenon in chronically abandoned leads. Indeed, this is the largest such study since Hartnell et al. in 1997 and echoes the seminal findings on this topic [15].

MRI is an important diagnostic modality for the post-cardiac surgery patient. Patients who have undergone cardiac surgery have a high rate of post-operative complications for which MRI is indicated such as stroke [22]. In the past, the presence of abandoned permanent or temporary epicardial pacing wires has been considered a relative contraindication for MRI [18, 23, 24]. However, current literature suggests that abandoned cardiovascular devices have a low incidence of associated adverse events. Hartnell et al. reported no major adverse events in 51 patients with abandoned TEPW who underwent MRI at 1–1.5T [15]. Gatterer et al. described minor event associated with TEPW, but their observations are limited to broad descriptors such as ‘C-shaped’ leads causing sensory changes during T2 HASTE sequences [25]. Recently a large meta-analysis of patients with retained cardiac devices included 129 patients with abandoned permanent epicardial pacing wire across 134 total MRI examinations. The authors found 5 (4.0%) minor events related to sensation in patients with retained permanent epicardial pacing wires and no major adverse events in chronically abandoned leads. However, the authors did note that there were changes in functional leads device impedance or parameter changes (*N* = 17, 13.2%) [16]. The rate of minor sensory complications reported in this study (0.9%) is compatible with the results from the mentioned meta-analysis (1%; 95% CI 0–2%), and with nearly the same number of scrutinized examinations. Additional studies related to permanent epicardial pacing wires have failed to identify consistent associated risk factors including device orientation or specific magnetic resonance sequences [9, 10]. Indeed, the authors reported no major MRI safety hazard related to chronically abandoned permanent epicardial pacing wires, similar to recent studies on 1.5T in abandoned permanent pacemaker/ICD leads [14].

It is important to recognize some of the demographic variables unique to this study. Only 5% of patients had an MRI in the first month after cardiac surgery. Many of the MRIs included in this study were for routine outpatient purposes. However, MRI is an important modality in the immediate post-operative period, especially for the workup of stroke [26]. Promisingly, a minority of patients underwent 3 T MRI with no reportable adverse events. While the number included remains small, further study is needed as 3 T MRI becomes more frequent in clinical practice.

There remain questions that are unanswered. Given that no single major event was recorded in 8 years, including in patients where the presence of abandoned TEPW was initially not known, major events must be a rare phenomenon. Although Balmer et al.‘s in vitro study suggested that epicardial leads not connected to a pacemaker, mimicking abandoned leads, may exhibit a more pronounced temperature rise at the lead tip, their findings did not translate into clinical practice [27]. It is unknown why there is this in-vitro to in-vivo discordance.

Our findings support American College of Radiology Manual on MR Safety 2024 Update specifically noting that no adverse outcomes have been reported to date in association with retained temporary epicardial wire fragments [8]. Given the theoretical possibility of major events, it is still worth discussing with the patient with known wires before their examination as routine MRI safety protocol. The authors recommend informing patients that there is a < 1% risk for a minor sensory phenomenon and a theoretical risk of more serious complication cannot be ruled out. In addition, a review of alternative diagnostic approaches should be considered. In the latter regard, it is advisable that ordering clinicians be included in these discussions, as they can offer a more nuanced rationale for the specific clinical rationale for MRI request. Nonetheless, it is likely that the benefits of MRI will outweigh the risk for many patients. When performing MRI in patients with retained TEPW, any update to manufacturer guidelines should be reviewed. If none are available, it would be reasonable to utilize institutional expertise to minimize unnecessary energy deposition.

The principal limitations of this study include the single institution protocolling and retrospective identification. The vast majority of TEPW were from a single manufacturer and a sizable number of patients were not able to have their brands identified because they were placed before 2016 and this information was not transferred to the new electronic medical record, or their procedure was performed elsewhere, and outside records were not available. It is conceivable that reactions in emergent patients with unknown TEPW could go unrecorded as the identification of patients relied on documentation in radiology reports, billing data, or clinical notes. For this reason, future prospective studies would benefit from multiple institutions contributing data which have different practices regarding their wire manufacturers. Additionally, pediatric patients were excluded from this study. However, there were a few adult congenital cardiac patients for which no events were recorded. Further study focusing on the pediatric population is reasonable. Lastly, there were a paucity of studies performed in the immediate post-operative period. It is reasonable to consider that there may be edema, post-operative fluid, or other factors which may influence rates of adverse events that were not captured in this study. A prospective analysis of patients in the first month post cardiac surgery would fill this gap in knowledge well.

## Conclusion

Abandoned TEPW demonstrate a < 1% rate of minor complications amongst adults undergoing MRI. No major adverse events were identified over the course of 8 years. MRI should not be withheld from patients with abandoned TEPW. Routine discussion of risks and benefits should continue with recognition that alternative tests may obviate the need for MRI. The low prevalence of minor adverse events is reassuring, and risks can be mitigated by ensuring compliance with manufacturer guidelines and minimizing energy deposition during imaging.

## Data Availability

The datasets generated and/or analyzed during the current study are not publicly available due protected health information but are available from the corresponding author on reasonable request.

## Abbreviations

• MRI: Magnetic Resonance Imaging
• TEPW: Temporary Epicardial Pacing Wires
• EACVI: European Association of Cardiovascular Imaging
• EHRA: European Heart Rhythm Association
• ESC: European Society of Cardiology
• CT: Computed Tomography
• CTA: Computed Tomography Angiography
• IQR: Interquartile Range
• T: Tesla
